# Combined effects of physical exercise and education on age-related cortical thinning in cognitively normal individuals

**DOI:** 10.1038/srep24284

**Published:** 2016-04-11

**Authors:** Jin San Lee, Hee Young Shin, Hee Jin Kim, Young Kyoung Jang, Na-Yeon Jung, Juyoun Lee, Yeo Jin Kim, Phillip Chun, Jin-Ju Yang, Jong-Min Lee, Mira Kang, Key-Chung Park, Duk L. Na, Sang Won Seo

**Affiliations:** 1 Department of Medicine, Graduate School, Kyung Hee University, Seoul, Korea; 2 Health Promotion Center, Samsung Medical Center, Sungkyunkwan University School of Medicine, Seoul, Korea; 3 Department of Neurology, Samsung Medical Center, Sungkyunkwan University School of Medicine, Seoul 06351, Korea; 4 Neuroscience Center, Samsung Medical Center 06351, Seoul, Korea; 5 Department of Neurology, Pusan National University Hospital, Pusan National University School of Medicine and Medical Research Institute, Busan, Korea; 6 Department of Neurology, Chungnam National University Hospital, Daejeon, Korea; 7 Department of Neurology, Chuncheon Sacred Heart Hospital, Hallym University College of Medicine, Chuncheon, Korea; 8 Department of Emergency Medicine Behavioral Emergencies Research Lab, San Diego, CA, USA; 9 Department of Biology, University of California San Diego, CA, USA; 10 Department of Biomedical Engineering, Hanyang University, Seoul, Korea; 11 Department of Neurology, Kyung Hee University School of Medicine, Seoul, Korea; 12 Department of Health Sciences and Technology, SAIHST, Sungkyunkwan University, Seoul 06351, Korea; 13 Department of Clinical Research Design & Evaluation, SAIHST, Sungkyunkwan University, Seoul 06351, Korea

## Abstract

We investigated the association between self-reported physical exercise and cortical thickness in a large sample of cognitively normal individuals. We also determined whether a combination of physical exercise and education had more protective effects on age-related cortical thinning than either parameter alone. A total of 1,842 participants were included in this analysis. Physical exercise was assessed using a questionnaire regarding intensity, frequency, and duration. Cortical thickness was measured using a surface-based method. Longer duration of exercise (≥1 hr/day), but not intensity or frequency, was associated with increased mean cortical thickness globally (*P*-value = 0.013) and in the frontal regions (*P*-value = 0.007). In particular, the association of exercise with cortical thinning had regional specificity in the bilateral dorsolateral prefrontal, precuneus, left postcentral, and inferior parietal regions. The combination of higher exercise level and higher education level showed greater global and frontal mean thickness than either parameter alone. Testing for a trend with the combination of high exercise level and high education level confirmed this finding (*P*-value = 0.001–0.003). Our findings suggest that combined exercise and education have important implications for brain health, especially considering the paucity of known protective factors for age-related cortical thinning.

Decline in cognitive function is associated with the normal aging process and has been termed ‘age-related cognitive decline.’ Although not fully understood, a large number of studies have revealed multiple risk factors contributing to age-related cognitive decline, including vascular risk factors/cardiovascular diseases^1^; inflammatory biomarkers^2^; malnutrition, especially vitamin B deficiency^3^; and lifestyle factors such as smoking, alcohol, and sleep problems^4^. However, there is also increasing evidence that lifestyle behaviors can mitigate age-related cognitive decline. Among these behaviors, education and physical exercise are generally accepted as protective factors against age-related cognitive decline.

Several studies have comprehensively described that education might increase cognitive reserve by increasing the number of neurons and synapses, which allows the brain to cope with age-related diseases using preexisting cognitive processes or enlisting compensatory approaches regardless of brain size^5^,^6^. Furthermore, in a recent study measuring cortical thickness, which is a promising marker of morphometric change in the brain during normal aging^7^, we demonstrated that higher level of education positively correlated with cortical thickness, and that these protective effects became more prominent with aging^8^.

Physical exercise is also known to have global effects on factors that influence general brain health^9^,^10^,^11^. There are several underlying mechanisms, including increased blood flow through vascularization and angiogenesis, activation of the immune system, and induction of neurotrophic factors. However, there have been few neuroimaging studies addressing the relationship between physical exercise and cortical thickness^12^,^13^,^14^. One previous study reported that higher level of physical exercise was associated with increased cortical thickness in the right prefrontal cortex^14^. However, these studies were conducted in selected and small patient samples. Moreover, there is a need to investigate which parameters of physical exercise, for example, intensity, frequency, or duration, have more beneficial effects on age-related cortical thinning. Considering the results of our previous study showing the effects of education on cortical thinning^8^, it would be reasonable to hypothesize that the combination of physical exercise and education would have more protective effects on age-related cortical thinning than either physical exercise or education alone.

In this study, we investigated the association between self-reported physical exercise and cortical thickness in a large sample of cognitively normal individuals. First, we explored which parameters of physical exercise –intensity, duration, or frequency, have more beneficial effects on age-related cortical thinning. Second, we determined whether a combination of physical exercise and education had more protective effects on age-related cortical thinning than either parameter alone.

## Results

### Comparisons of baseline characteristics according to exercise parameters

Table 1 summarizes the baseline characteristics of the 1,842 participants. In the study population, the mean (SD) age was 63.8 (6.9) years, ranging from 45 to 91 years; and the mean (SD) education length was 12.9 (4.2) years. In addition, the distributions of the dichotomized exercise groups were as follows: 681 (37.0%) patients were in the longer duration group (≥1 hr/day), 495 (26.9%) patients were in the higher intensity group (≥moderate intensity), and 549 (29.8%) patients were in the higher frequency group (≥5 days/week).

There were differences in demographics and/or vascular risk factors between the subgroups with higher exercise parameters and those with lower exercise parameters: education and diabetes mellitus varied by exercise duration; age, sex, education, ischemic heart disease, familial history of stroke, height, weight, and ICV varied by exercise intensity; age, education, history of stroke, height, weight, and K-MMSE varied by exercise frequency (Table 1 and Supplementary Table 1).

### Relationships between exercise parameters and cortical thickness

In Model 1, subjects with longer durations of exercise had greater cortical thickness in the global and frontal regions than those with shorter durations (Table 2). On the other hand, there was no significant relationship between intensity or frequency of exercise and cortical thickness. When we simultaneously added the three exercise parameters to the independent variables in Model 2, longer exercise duration, but not intensity or frequency, was associated with cortical thickness globally (*P*-value = 0.013) and in the frontal regions (*P*-value = 0.007, Table 2). In sensitivity analyses, we performed additional analyses after excluding 140 participants who had a history of ischemic heart disease or stroke (Total N = 1,702). The new results were similar with previous one (Supplementary Table 2).

**Figure 1:**
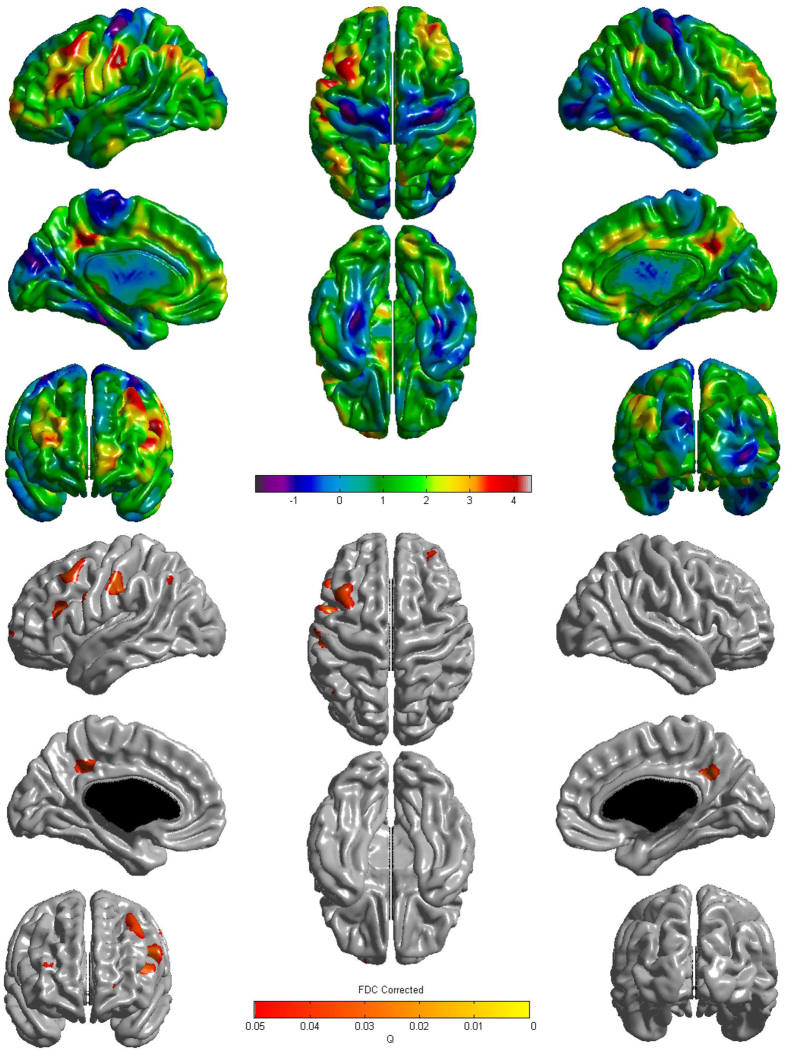
Three-dimensional reconstruction for correlation between physical exercise and cortical thickness. The association of longer duration of exercise with cortical thinning had regional specificity in the bilateral dorsolateral prefrontal, precuneus, left postcentral, and inferior parietal regions. The Q value denotes the FDR-corrected *P* value.

Application of the general linear model to vertex-wise cortical thickness showed that, in comparison to the shorter exercise duration group, the longer exercise duration group exhibited greater cortical thickness in the bilateral dorsolateral prefrontal, precuneus, left postcentral, and inferior parietal regions (Fig. 1).

### Relationships between duration of physical exercise, education level, and cortical thickness

**Figure 2:**
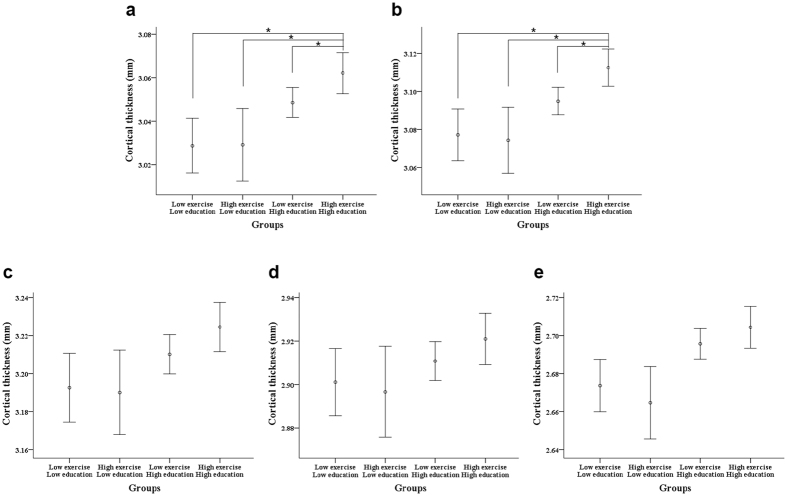
Mean cortical thickness in four groups was classified according to exercise and education. (**a**) Global region, (**b**) Frontal region, (**c**) Temporal region, (**d**) Parietal region, (**e**) Occipital region. ‘o’ denotes mean cortical thickness, error bars shows the 95% confidence interval (CI). *P < 0.05.

Figure 2 revealed that the group with a combination of higher exercise and higher education had greater global and frontal mean thickness than groups with only higher exercise, higher education, or lower exercise and lower education. Tests for linear trends across the combined exercise and education effects showed that combined higher exercise and higher education was associated with increased mean cortical thickness globally (p for linear trends = 0.003), and in the frontal region (p for linear trends = 0.001, Fig. 2).

**Figure 3:**
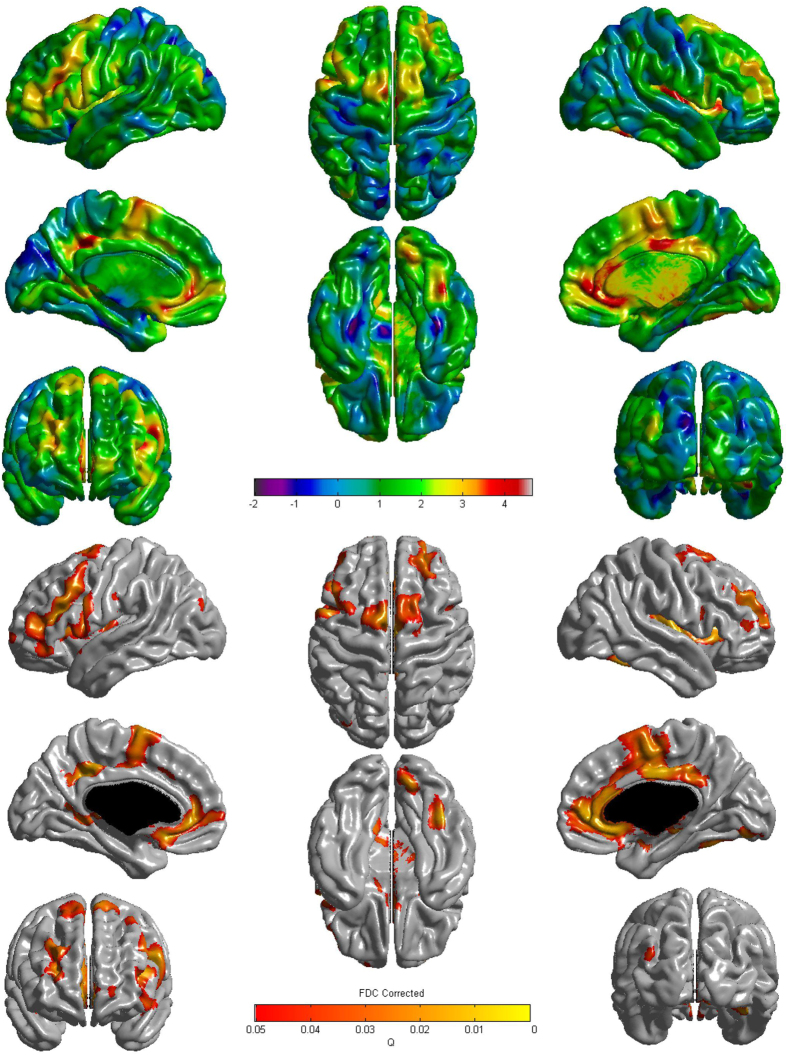
Regional differences in cortical thickness between the group with higher educatation level and higher exercise duration and the group with lower education level and lower exercise duration. The association of lower education level and lower exercise duration with cortical thinning had more extensive regional specificity in the bilateral dorsolateral prefrontal, supplementary motor, anterior and posterior cingulate, precuneus, and insular regions. The Q value denotes the FDR-corrected *P* value.

Regional differences in cortical thickness in patients with longer exercise duration (≥1 hr/day) and higher education (≥12yrs) were reflected as greater thickness in the anterior and posterior cingulate, insular, and supplementary motor regions, as well as in the bilateral dorsolateral prefrontal, precuneus, and inferior parietal regions, compared with the groups with shorter exercise duration (<1 hr/day) and lower education (<12yrs, Fig. 3).

## Discussion

We report new evidence of a relationship among exercise, education, and cortical thickness in cognitively normal individuals. Our major findings were as follows. First, longer duration of exercise (≥1 hr/day), but not the frequency or intensity of exercise, was correlated with increased cortical thickness in the bilateral dorsolateral prefrontal cortex, precuneus, left postcentral gyrus, and inferior parietal regions. Second, a combination of higher exercise and higher education was associated with greater global and frontal mean thickness than either exercise or education alone. Furthermore, tests for trends across combinations of high exercise and high education confirmed this finding. Taken together, our findings suggest that combined exercise and education has important implications in brain health, especially considering the paucity of known protective factors for age-related cortical thinning.

Our first major finding was that longer duration of exercise (≥1 hr/day), but not intensity or frequency, was significantly associated with increased mean cortical thickness globally and in the frontal regions. Furthermore, the effects of longer duration of exercise remained significant after adjustments for the other two exercise parameters (intensity and frequency). To the best of our knowledge, the question of which exercise parameters have more beneficial effects on age-related cortical thinning remains unsettled^15^,^16^,^17^,^18^. A previous study suggested that moderate-intensity exercise was associated with better cognitive performance regardless of frequency^15^, while another study showed that the beneficial effects of physical exercise on memory were independent of exercise intensity^16^. Several studies have also suggested that the amount of exercise is related with GM volume^17^,^18^, although they did not discriminate the effects of duration or frequency of exercise. Therefore, our findings suggest that the duration of exercise is a more influential parameter on cortical thickness than are other parameters such as frequency and intensity.

Most regional differences in cortical thinning related to longer exercise duration were noted in the bilateral dorsolateral prefrontal precuneus and inferior parietal regions. Our findings were generally consistent with previous studies showing that increased exercise was associated with increased GM volume in the prefrontal and posterior cingulate regions^14^,^16^,^17^. The dorsolateral prefrontal region is a key region of the brain associated with executive functions such as working memory, cognitive flexibility, and planning^19^,^20^,^21^. In addition, the precuneus has a central role in highly integrated tasks, including episodic memory retrieval, visuospatial processing, and self-consciousness^22^. The inferior parietal region is known to play a key role in various cognitive functions such as attention, language, and visuospatial and action-related functions^23^,^24^. These regions are also known to be susceptible to age-related deterioration or Alzheimer’s disease (AD)^10^,^25^,^26^,^27^. Therefore, our results suggest that physical exercise is associated with protective effects on age- or AD-related cortical thinning.

Our second major finding was that a combination of higher exercise and higher education had greater effects on mean thickness globally and in the frontal regions than did either parameter alone. Tests for trends across combinations of high exercise and high education confirmed this finding. To the best of our knowledge, there have been no studies investigating the combined effects of education and exercise on cortical thinning. However, our findings are supported by a few randomized controlled trials that have suggested that a combination of education and physical exercise more highly enhanced cognitive function than did either parameter alone^28^,^29^,^30^. Increased cortical thickness related to the combination of exercise and education was observed more frequently than the increased thickness observed with exercise. In particular, increased thickness of the anterior and posterior cingulate, insular, and supplementary motor regions are reported to be related to education level^31^,^32^. The mechanisms through which the combined effects of physical exercise and education on age-related cortical thinning become greater than those of either parameter alone are unknown. However, the effects of education are known to be mediated by increased neural reserve^5^, while the effects of exercise might be explained by vascularization or angiogenesis^10^,^11^. Previous studies have shown that neurovascular decoupling is also an important mechanism that can explain age-related cognitive impairment or AD^33^. It is therefore reasonable to suggest that exercise and education increase vascularization and neural reserve, which in turn lead to enhanced neurovascular coupling, eventually resulting in more beneficial effects than either parameter alone.

The strengths of this study include the large sample size and sophisticated measurements of cortical thickness. However, some limitations should be considered when interpreting the results. First, we used self-reported physical exercise in the questionnaire; hence, recall bias is inherent. Interventional studies would help to more precisely assess physical exercise. Second, our participants were recruited from individuals seeking a comprehensive preventive health exam not covered by national medical insurance, which might limit the generalizability of this study to the general population. Third, the association between duration of exercise and cortical thickness is modest, but the association remained even when other risk factors known to affect brain structures were included in models. Finally, our study was designed to be cross-sectional, precluding claims of causality. Nevertheless, it is noteworthy that this is the first study showing the combined effects of education and exercise on age-related cortical thinning.

## Methods

### Study participants

We studied 2,217 participants who attended a preventative medical check-up, which included an assessment of cognitive function and dementia status, at the Health Promotion Center of the Samsung Medical Center (Seoul, Korea) from July 2009 to December 2014. Of these participants, 1,959 have been described in a previous study on the effects of education on cortical thinning^8^. All study participants underwent brain magnetic resonance imaging (MRI), including three-dimensional volume images, as a part of their dementia assessment. We excluded the following participants from this study: 16 participants who were younger than 45 years of age; 88 participants with significant cognitive impairment defined by Mini-Mental Status Examination (MMSE) scores below the 16th percentile in age-, sex-, and education-matched norms or through an interview conducted by a qualified neurologist; and 227 participants with missing data on demographics (N = 189), anthropometric variables (N = 9), or history of hypertension (N = 14), diabetes mellitus (N = 5), or hyperlipidemia (N = 10). We also excluded 44 participants with unreliable analyses of cortical thickness due to head motion, blurring of the MRI, inadequate registration to a standardized stereotaxic space, misclassification of tissue type, or inexact surface extraction. The final sample size was composed of 1,842 participants. Apolipoprotein E (APOE) genotyping was performed in 438 (23.8%) of the 1,842 participants in this study.

### Standard protocol approvals, registrations, and patient consents

This study was approved by the Institutional Review Board at Samsung Medical Center. In addition, all methods were carried out in accordance with the approved guidelines. The requirement for participant’s consent was waived since we used retrospective de-identified data collected during health exam visits.

### Measurements

Health screening exams were conducted by trained personnel according to the standard protocol. Health screening exams included a questionnaire, physical exam, laboratory analyses, and brain MRI. Information regarding this screening program has been previously described in detail^34^. The questionnaire data included questions on medical history and medication use. To precisely evaluate the level of formal education achieved by the participants, we collected information on completed education (elementary school, middle school, high school, college, and graduate school) and the total duration of education.

Hypertension was defined as systolic blood pressure (SBP) ≥140 mmHg, diastolic blood pressure (DBP) ≥90 mmHg, or use of antihypertensive medication. Quality control procedures were performed in accordance with the Korean Association of Laboratory Quality Control. A patient was considered to have diabetes mellitus if he or she was prescribed diabetes medication or had a fasting blooding sugar level ≥126 mg/dl.

### Physical exercise questionnaire

Physical exercise was assessed using the modified Korean version of International Physical Activity Questionnaire 7 (IPAQ-7)^35^,^36^,^37^. The questionnaire was a subjective measure that asked participants to recall their physical activity from the previous 7 days. It was separated into 3 sections and consisted of (1) the intensity (vigorous/moderate/light/very light/none), (2) the frequency (per week, more than 5 days/3–4 days/1–2 days/none), and (3) the duration (per day, more than 60 min/40–60 min/20–40 min/less than 20 min) of physical activity. Exercise intensity was presented in terms of the following activities; vigorous (e.g., aerobic exercise or playing soccer), moderate (e.g., cycling or mountain climbing), light (e.g., walking more than 10 min or doing housework), and very light or none (e.g., walking less than 10 min or no exercise).

For analysis, we dichotomized all subscales of the questionnaire following the current Physical Activity Guidelines for Adults^38^ which is moderate-intensity aerobic physical activity to 300 minutes (5 hours) each week. Then we used the cut-off, one hour per day, 5 days per week, and moderate intensity. Finally, three exercise parameters were created based on predefined variables: an intensity group (dichotomized as “higher” vs. “lower” based on moderate intensity), a frequency group (dichotomized as “higher” vs. “lower” based on 5 days per week), and a duration group (dichotomized as “longer” vs. “shorter” based on one hour per day).

### Brain MRI scans

All participants underwent neurological and neuropsychological examination, MMSE, and 3D volumetric brain MRI scan. An Achieva 3.0-Tesla MRI scanner (Philips, Best, the Netherlands) was used to acquire 3D T1 Turbo Field Echo (TFE) MRI data from 2,310 participants using the following imaging parameters: sagittal slice thickness, 1.0 mm with 50% overlap; no gap; repetition time of 9.9 ms; echo time of 4.6 ms; flip angle of 8°; and matrix size of 240 × 240 pixels reconstructed to 480 × 480 over a field view of 240 mm.

Radiologists initially inspected all MRI images for evidence of brain tumors, lobar infarctions (except lacunar infarctions), and hemorrhages (observed as low intensity areas in T2-weighted images).

T1-weighted MR images were automatically processed using the standard Montreal Neurological Institute image processing software (CIVET) to measure cortical thickness. The software has been well-validated and extensively described elsewhere including aging/atrophied brain studies^39^,^40^,^41^,^42^,^43^. In summary, native MRI images were first registered into a standardized stereotaxic space using an affine transformation^44^. Non-uniformity artifacts were corrected using the N3 algorithm, and the registered and corrected volumes were classified as white matter, gray matter (GM), cerebrospinal fluid, and background using an artificial neural net classifier^45^. The surfaces of the inner and outer cortices were automatically extracted by deforming a spherical mesh onto the gray/white boundary of each hemisphere using the Constrained Laplacian-Based Automated Segmentation with Proximities algorithm, which has also been well-validated and extensively described elsewhere^46^,^47^.

Cortical thickness was calculated as the Euclidean distance between the linked vertices of the inner and outer surfaces after applying an inverse transformation matrix to cortical surfaces and reconstructing them in the native space^47^,^48^. To control for brain size, we computed ICV using classified tissue information and a skull mask acquired from the T1-weighted image^49^. ICV was defined as total volume of GM, white matter (WM), and cerebrospinal fluid (CSF), with consideration of voxel dimension. Classified GM, WM, CSF, and background within the mask were transformed back into individual native space.

To compare the thicknesses of corresponding regions among the participants, the thicknesses were spatially registered on an unbiased iterative group template by matching the sulcal folding pattern using surface-based registration involving sphere-to-sphere warping^50^,^51^. For global and lobar regional analyses, we used the lobe-parcellated group template that had been previously divided into frontal, temporal, parietal, and occipital lobes using SUMA (http://afni.nimh.nih.gov)^48^. Average values of thickness of the whole vertex in each hemisphere and lobar region were used for global analysis.

### Statistical analysis

For demographic comparison, Chi-square and *t*-tests were performed between two groups. To evaluate the relationship of physical exercise and cortical thickness, we used multiple linear regression analysis models. In Model 1, the three exercise parameters were analyzed separately with regard to mean cortical thickness after controlling for age, sex, education (continuous), ICV, vascular risk factors (hypertension, diabetes mellitus, hyperlipidemia, and BMI), ischemic heart disease, and history of stroke. Model 2 was further adjusted for the two exercise parameters not used in the analysis.

In order to determine whether the combination of higher exercise and higher education had more beneficial effects on age-related cortical thinning than only higher exercise or higher education, we stratified our subjects into four groups: lower exercise (<1 hr/day) and lower education (<12yrs) (N = 256), higher exercise (≥1 hr/day) and lower education (<12yrs) (N = 184), lower exercise (<1 hr/day) and higher education (≥12yrs) (N = 905), and higher exercise (≥1 hr/day) and higher education (≥12yrs) (N = 497). In order to evaluate tests for linear trends across the combined physical exercise and education groups, we also entered four groups of exercise and education as continuous variables into Model 2. SPSS 20 (SPSS Inc., Chicago, IL, USA) was used for all statistical analyses.

For cortical thickness analyses, we used a MATLAB-based toolbox (available free online at the University of Chicago website: http://galton.uchicago.edu/faculty/InMemoriam/worsley/research/surfstat/). Diffusion smoothing with a full-width half-maximum of 20 mm was used to blur each cortical thickness map, leading to increased signal-to-noise ratio and statistical power^40^. In order to analyze the localized differences and the statistical map of cortical thickness on the surface model, linear regression was performed vertex-by-vertex after controlling for age, sex, education, ICV, vascular risk factors, ischemic heart disease, history of stroke, and the two exercise parameters not used in the analysis. The resulting statistical maps were thresholded using a false discovery rate (FDR)^52^ with a q value of 0.05 after pooling the *P*-values from regressions analysis.

## Additional Information

**How to cite this article**: Lee, J. S. *et al.* Combined effects of physical exercise and education on age-related cortical thinning in cognitively normal individuals. *Sci. Rep.* **6**, 24284; doi: 10.1038/srep24284 (2016).

## Supplementary Material

Supplementary Information

## Figures and Tables

**Table 1:** Distribution of self-reported assessment of physical exercise and comparisons of the characteristics between the exercise duration groups.

	Total	Exercise group		
		Longer duration (≥1 hr/day)	Shorter duration (<1 hr/day)	*P*-value
Number (%)	1842 (100)	681 (37.0)	1161 (63.0)	
Age, years	63.8 (6.9)	64.1 (6.5)	63.7 (7.2)	0.213
Female, N (%)	858 (46.6)	321 (47.1)	537 (46.3)	0.714
Education, years	12.9 (4.2)	12.6 (4.4)	13.1 (4.1)	0.022*
Hypertension, N (%)	847 (46.0)	302 (44.3)	545 (46.9)	0.281
Diabetes mellitus, N (%)	308 (16.7)	97 (14.2)	211 (18.2)	0.029*
Hyperlipidemia, N (%)	608 (33.0)	219 (32.2)	389 (33.5)	0.553
Ischemic heart disease, N (%)	103 (5.6)	29 (4.3)	74 (6.4)	0.056
History of stroke, N (%)	43 (2.3)	20 (2.9)	23 (2.0)	0.190
Familial history of stroke, N (%)	397 (21.6)	148 (21.7)	249 (21.4)	0.886
Familial history of dementia, N (%)	266 (14.4)	95 (14.0)	171 (14.7)	0.646
BMI, kg/m2	23.9 (2.6)	23.8 (2.5)	24.0 (2.7)	0.143
Height, cm	162.7 (8.1)	162.4 (8.2)	162.9 (8.1)	0.208
Weight, kg	63.6 (9.8)	63.0 (9.5)	63.9 (9.9)	0.060
ICV, cm3	1354.6 (123.5)	1354.1 (119.9)	1355.0 (125.6)	0.882
K-MMSE, points	28.1 (1.8)	28.1 (1.8)	28.1 (1.8)	0.481
Exercise parameters				
Higher intensity, N (%)	495 (26.9)	259 (52.3)	236 (47.7)	
Higher frequency, N (%)	549 (29.8)	322 (58.7)	227 (41.3)	
Longer duration, N (%)	681 (37.0)			

Chi-square and t-tests were performed to compare demographic variables between two exercise groups. Values are mean (SD) or number (%).

N: number, SD: standard deviation, BMI: body mass index, ICV: intracranial volume, K-MMSE: Korean mini mental status examination, *P < 0.05.

**Table 2:** Relationships between exercise parameters and mean cortical thickness.

	Global			Frontal			Temporal			Parietal			Occipital		
	*B*	*SE*	*p*	*B*	*SE*	*p*	*B*	*SE*	*p*	*B*	*SE*	*p*	*B*	*SE*	*p*
Model 1															
Duration	0.011	0.005	0.023*	0.013	0.005	0.011*	0.010	0.007	0.168	0.008	0.006	0.192	0.006	0.006	0.305
Intensity	−0.002	0.005	0.752	0.001	0.006	0.929	0.002	0.008	0.778	−0.001	0.007	0.847	−0.010	0.006	0.118
Frequency	<0.001	0.005	0.970	<0.001	0.006	0.976	0.013	0.008	0.095	−0.005	0.007	0.490	−0.006	0.006	0.342
Model 2															
Duration	0.013	0.005	0.013*	0.015	0.006	0.007*	0.007	0.008	0.349	0.011	0.007	0.107	0.010	0.006	0.102
Intensity	−0.004	0.006	0.458	−0.002	0.006	0.703	<0.001	0.008	0.958	−0.003	0.007	0.688	−0.011	0.006	0.082
Frequency	−0.004	0.006	0.497	−0.004	0.006	0.479	0.011	0.008	0.183	−0.008	0.007	0.277	−0.008	0.006	0.226

Model 1, multiple linear regressions were performed after adjusted for age, sex, education (continuous), history of hypertension, diabetes mellitus, hyperlipidemia, ischemic heart disease, stroke, BMI, and ICV.

Model 2, multiple linear regressions were performed after further adjusted for the two exercise parameters not used in the analysis.

*B (SE)*: β value (standard error of the mean), *P < 0.05.
