# Supplementary material for: Combined effects of physical exercise and education on age-related cortical thinning in cognitively normal individuals
Source: Sci Rep. 2016 Apr 11;6:24284. doi: 10.1038/srep24284 (PMC4827124; doi:10.1038/srep24284)
Supplement: Supplementary Information [file srep24284-s1.pdf]

Combined effects of physical exercise and education on age-related cortical thinning in cognitively normal individuals

Jin San Lee, MD,[1,2] Hee Young Shin, MD,[3] Juyoun Lee, MD,[1,2] Young Kyoung Jang, MD,[1,2] Na-Yeon Jung, MD,[4] Phillip Chun,[5,6] Jin-Ju Yang, MS,[7] Jong-Min Lee, PhD,[7] Mira Kang, MD,[3] Yeo Jin Kim, MD,[1,2] Hee Jin Kim, MD,[1,2] Key-Chung Park, MD,[8] Duk L. Na, MD,[1,2,9] Sang Won Seo, MD[1,2,9,10]

[1] Department of Neurology, Samsung Medical Center, Sungkyunkwan University School of Medicine, Seoul 06351, Korea

[2] Neuroscience Center, Samsung Medical Center 06351, Seoul, Korea

[3] Health Promotion Center, Samsung Medical Center, Sungkyunkwan University School of Medicine, Seoul, Korea

[4] Department of Neurology, Pusan National University Hospital, Pusan National University School of Medicine and Medical Research Institute, Busan, Korea

[5] Department of Emergency Medicine Behavioral Emergencies Research Lab, San Diego, CA, USA

[6] Department of Biology, University of California San Diego, CA, USA

[7] Department of Biomedical Engineering, Hanyang University, Seoul, Korea

[8] Department of Neurology, Kyung Hee University School of Medicine, Seoul, Korea

[9] Department of Health Sciences and Technology, SAIHST, Sungkyunkwan University, Seoul 06351, Korea

[10] Department of Clinical Research Design & Evaluation, SAIHST, Sungkyunkwan University, Seoul 06351, Korea

Corresponding author

Sang Won Seo, MD, PhD

Department of Neurology, Samsung Medical Center, Sungkyunkwan University School of Medicine, 81 Irwon-ro, Kangnam-ku, Seoul 06351, Korea

Tel: +82-2-3410-1233, Fax: +82-2-3410-0052, E-mail address: sangwonseo@empal.com

Supplementary data: Supplementary Table 1 and Supplementary Table 2

Supplementary Table 1. Comparison of the characteristics between the exercise groups according to exercise parameters

	Intensity group[a]			Frequency group[b]		
	**Higher intensity**	**Lower intensity**	***P*-value**	**Higher frequency**	**Lower frequency**	***P*-value**
Total, N (%)	495 (26.9)	1347 (73.1)		549 (29.8)	1293 (70.2)	
Age, years	63.3 (6.3)	64.0 (7.1)	0.027*	65.2 (7.2)	63.3 (6.7)	<0.001*
Female, N (%)	186 (37.6)	672 (49.9)	<0.001*	266 (48.5)	592 (45.8)	0.294
Education, years	13.4 (4.3)	12.7 (4.1)	0.001*	12.1 (4.7)	13.3 (3.9)	<0.001*
Hypertension, N (%)	227 (45.9)	620 (46.0)	0.948	266 (48.5)	581 (44.9)	0.166
Diabetes mellitus, N (%)	69 (13.9)	239 (17.7)	0.052	88 (16.0)	220 (17.0)	0.604
Hyperlipidemia, N (%)	167 (33.7)	441 (32.7)	0.686	176 (32.1)	432 (33.4)	0.572
Ischemic heart disease, N (%)	19 (3.8)	84 (6.2)	0.047*	33 (6.0)	70 (5.4)	0.610
History of stroke, N (%)	11 (2.2)	32 (2.4)	0.847	20 (3.6)	23 (1.8)	0.015*
Familial history of stroke, N (%)	123 (24.8)	274 (20.3)	0.037*	112 (20.4)	285 (22.0)	0.433
Familial history of dementia, N (%)	74 (14.9)	192 (14.3)	0.707	77 (14.0)	189 (14.6)	0.741
BMI, kg/m2	24.0 (2.4)	23.9 (2.7)	0.797	23.7 (2.7)	24.0 (2.6)	0.041
Height, cm	164.0 (8.1)	162.3 (8.1)	<0.001*	161.9 (8.4)	163.1 (8.0)	0.003*
Weight, kg	64.7 (9.4)	63.2 (9.9)	0.003*	62.3 (9.7)	64.1 (9.7)	0.001*
ICV, cm3	1369.7 (123.8)	1349.1 (123.0)	0.001*	1352.1 (122.9)	1355.7 (123.8)	0.569
K-MMSE, points	28.2 (1.7)	28.1 (1.8)	0.114	27.8 (2.0)	28.2 (1.6)	<0.001*
Mean cortical thickness, mm						
Global	3.051 (0.106)	3.046 (0.107)	0.829	3.041 (0.109)	3.051 (0.106)	0.926
Frontal	3.099 (0.112)	3.094 (0.113)	0.842	3.088 (0.114)	3.098 (0.112)	0.984
Temporal	3.215 (0.151)	3.208 (0.155)	0.695	3.212 (0.151)	3.208 (0.155)	0.105
Parietal	2.913 (0.138)	2.910 (0.135)	0.877	2.901 (0.132)	2.915 (0.137)	0.472
Occipital	2.691 (0.123)	2.692 (0.124)	0.130	2.681 (0.129)	2.697 (0.121)	0.310

[a] The exercise groups were defined as higher and lower according to intensity of exercise (vigorous and moderate intensity vs. light and very light intensity or no exercise).
[b] The exercise groups were defined as high and low according to frequency of exercise per week (more than 5 days vs. less than 5 days and no exercise).
Chi-square and t-tests were performed to compare demographic variables between two exercise groups. ANCOVA was used to compare mean cortical thickness between two groups (*$P < 0.05$). Values are mean (SD) or number (%). N: number, SD: standard deviation, BMI: body mass index, ICV: intracranial volume, K-MMSE: Korean mini mental status examination, * $P < 0.05$

Supplementary Table 2. Relationships between exercise parameters and mean cortical thickness (N = 1,702)

	**Global**			**Frontal**			**Temporal**			**Parietal**			**Occipital**		
	B	***SE***	***p***	***B***	***SE***	***p***	***B***	***SE***	***p***	***B***	***SE***	***p***	***B***	***SE***	***p***
Model 1															
Duration	0.012	0.005	0.019*	0.014	0.005	0.008*	0.011	0.007	0.147	0.009	0.006	0.169	0.006	0.006	0.315
Intensity	-0.001	0.005	0.843	0.001	0.006	0.850	0.004	0.008	0.616	-0.002	0.007	0.815	-0.010	0.006	0.124
Frequency	<0.001	0.005	0.937	<0.001	0.006	0.993	0.013	0.008	0.108	-0.005	0.007	0.505	-0.006	0.006	0.327
Model 2															
Duration	0.013	0.005	0.011*	0.016	0.006	0.006*	0.008	0.008	0.323	0.011	0.007	0.091	0.010	0.006	0.104
Intensity	-0.004	0.006	0.523	-0.002	0.006	0.763	0.001	0.008	0.874	-0.003	0.007	0.644	-0.011	0.006	0.087
Frequency	-0.004	0.006	0.450	-0.005	0.006	0.432	0.010	0.008	0.217	-0.008	0.007	0.278	-0.008	0.006	0.215

Model 1, multiple linear regressions were performed after adjusted for age, sex, education (continuous), history of hypertension, diabetes mellitus, hyperlipidemia, BMI, and ICV.
Model 2, multiple linear regressions were performed after further adjusted for the two exercise parameters not used in the analysis.
B (SE): β value (standard error of the mean), *P < 0.05
